# The impact of balance exercise on brain age and brain morphometry: insights from MRI analysis

**DOI:** 10.1007/s40520-026-03322-6

**Published:** 2026-01-22

**Authors:** Varima Narula, Denise Taylor, Ruth McLaren, Rachael L. Taylor, Susan Mahon, Paul F. Smith, Shikha Chaudhary, Roger W. Winton, Justin Fernandez, Vickie Shim, Alan Wang

**Affiliations:** 1 Auckland Bioengineering Institute, The University of Auckland, Auckland, New Zealand; 2 Health and Rehabilitation Research Institute, Auckland University of Technology, Auckland, New Zealand; 3 Department of Physiology and Centre for Brain Research, Faculty of Medical and Health Sciences, University of Auckland, Auckland, New Zealand; 4 Traumatic Brain Injury Network, Auckland University of Technology, Northcote, Auckland, New Zealand; 5 Department of Pharmacology and Toxicology, Faculty of Biomedical and Molecular Sciences, University of Otago, Dunedin, New Zealand; 6 Faculty of Medical and Health Sciences, Eisdell Moore Centre for Hearing and Balance Research, University of Auckland, Auckland, New Zealand; 7 Department of Engineering Science and Biomedical Engineering, University of Auckland, Auckland, New Zealand; 8 Medical Imaging Research Centre, Faculty of Medical and Health Sciences, The University of Auckland, Auckland, New Zealand; 9 Centre for Co-Created Ageing Research, The University of Auckland, Auckland, New Zealand; 10 Centre for Brain Research, The University of Auckland, Auckland Bioengineering House, 70 Symonds Street, Auckland, 1010 New Zealand

**Keywords:** Balance exercise, Brain age, Brain volume, Freesurfer, Brain MRI

## Abstract

Physical exercise is known to delay the cognitive decline in the elderly. However, the effect of low-impact balance exercises such as yoga or Tai chi has not been explored in detail. This cross-sectional observational study used brain magnetic resonance imaging data to quantify and compare various brain structures between neurologically healthy adults aged between 55 and 65, divided into Control Group and Balance Exercise (BE) Group based on the self-reported balance exercise status. Various brain attributes such as brain age, cortical and subcortical volume, thickness, surface area, and mean curvature were extracted and computed using machine learning algorithm software like brainageR and FreeSurfer. Clinical functional assessments (balance, vestibular and cognitive measures) were also conducted for the participants. Statistical analyses were performed to determine any differences between the groups at a significance level of 5%. The BE group showed statistically significantly higher values for the right caudal anterior cingulate thickness, left and right superior temporal volume, left entorhinal volume and mean curvature, left frontal pole thickness, left superior temporal area and left inferior temporal thickness. A statistically significant cluster after correction for multiple comparisons was found in the left rostral middle frontal gyrus with a higher volume for BE group. Clinical functional assessments (balance, vestibular and cognitive) and brain age differences were nonsignificant. The significant brain regions in the BE group are involved in memory, cognition, focus, planning, language and auditory processing, decision making, emotional regulation and mental health and could be responsible for protecting and delaying the cognitive declines in the elderly.

**Supplementary Information:**

The online version contains supplementary material available at 10.1007/s40520-026-03322-6.

## Introduction

According to the World Health Organization it is expected that the worldwide population of people aged 60 years or over will increase from 1 billion in 2020 to 1.4 billion in 2030 and double by 2050 to 2.1 billion; and the number of individuals aged 80 years or older is expected to triple between 2020 and 2050, reaching 426 million [1]. Aging is associated with declining motor and cognitive abilities [2]. As a response to the challenges of an aging world-wide population the United Nations declared 2021–2030 as the Decade of Healthy Ageing, aiming to improve the lives of families, and communities through minimising some of the negative effects of aging [1, 3, 4].

The process of ageing is complex, involving multiple factors and impacting various organs and systems in the body [5]. There is heterogeneity in the ageing processes not only between individuals but also within an individual [6]. Cardiovascular exercise has been shown to protect against some of the effects of aging, reducing mortality and morbidity [7], increasing brain volumes [8], and delaying decline in cognitive function [9]. To our knowledge, no research has investigated whether balance exercise has a similar effect. Three-dimensional balance exercise such as Tai Chi, yoga and dance are practiced by millions of people worldwide [10, 11]. They are reported to improve balance and mobility [12, 13], particularly targeting physical strength, flexibility and postural control [11]. However, little is known about the effect of long-term balance exercise on the brain and healthy aging. Advances in neuroimaging now give us the opportunity to investigate this.

### Healthy aging

Several novel methodologies have been developed for estimating an individual’s “biological age” to compare with the chronological age [6, 14]. It is a known fact that the brain changes in its structure and cognitive abilities as we age [9, 15]. Changes in the brain associated with the normal ageing process can be measured using machine-learning algorithms on neuroimaging data and used to predict the chronological age of an individual quite precisely [7, 16, 17]. A standardised ‘brain-age’ calculation model can be used to determine large deviations from the chronological age which can indicate divergence from normal ageing [14, 18]. Individuals with cognitive or neurological disorders have been shown to have a higher brain age as compared to their chronological age: such as in Alzheimer’s disease [8], depression [19], schizophrenia [20], and bipolar disorder [21]. On the other hand, individuals with healthy lifestyle choices such as years of education, a high amount of physical activity [22] and practice of long-term meditation [23], have been shown to have younger brains.

### Neuroimaging

Neuroimaging studies the brain structure and function in a non-invasive way, using computational and quantitative techniques, and is an essential tool for diagnostic procedures and research studies [24]. Neuroimaging techniques allow us to visualize the brain’s structure, its electrical activity and neural network connections [24]. It has enhanced our understanding of brain structural organisation, the functional involvement of different regions of the brain in various behavioural and mental processes, and neuroplasticity [25, 26].

Structural imaging is one of the main categories for neuroimaging studies and it is used to study the brain structures. Magnetic Resonance Imaging (MRI) is one of the main modes for structural neuroimaging because its superior image quality with excellent soft tissue contrasts and high spatial resolution provides an excellent diagnostic value [27]. Structural MRI like T1 weighted image is a great tool for analysing the volumetric and morphometric changes in the brain [28].

The neural mechanisms that are influenced by physical exercise, especially exercises that focus on balance and coordination, in regard to healthy ageing, have not been explored in great detail. The neurological adaptations or modifications brought about by balance exercises and their prospective role in protecting cognitive function and overall health of ageing individuals needs to be investigated. The brain structures that are associated with balance, vestibular and cognitive functions need to be quantified to elucidate if there are any structural changes associated with doing balance exercise in aging brains.

The aim of this study was to examine the relationship between long term 3-dimensional balance exercise and brain anatomy, alongside clinical assessments for balance, vestibular function and cognitive measures that can represent healthy aging. We hypothesized that individuals who do balance exercise would perform better in the clinical assessments and have younger brains with larger volumes than individuals who do not. The comparison of different brain attributes will provide an insight into the impact of balance exercise on neurological changes in healthy aging.

## Methods

### Participants

Adults between the age of 55–65 were recruited in this cross-sectional observational study. Inclusion in the balance exercise group required participants to self-report participating in balance exercise (Tai chi, yoga, Pilates, surfing, dance or a group exercise class with a significant balance component) at least one hour a week, most weeks, for the previous 5 years. The control group had not partaken in any balance exercise over the previous 5 years, and their weekly exercise in the previous year fell below the recommended exercise guidelines for older adults (< 150 min of moderate exercise, < 60 min of vigorous exercise and < 60 min of muscle strengthening exercise a week). Participants were excluded if they had been diagnosed with a neurological condition or had contraindications for MRI. Individuals who had participated in any formal exercise for more than 12 weeks in the previous year were excluded from the control group. Self-reported activity levels were collected using the New Zealand physical activity questionnaire, a reliable measure of activity levels validated in a New Zealand population [29]. This study was approved by Auckland University of Technology Ethics Committee (AUTEC reference: 21/123).

### Clinical assessments

#### Balance

Participants completed the modified clinical test of sensory integration in balance (m-CTSIB) for balance assessment. m-CTSIB measures *postural stability* in four conditions: standing on Firm ground with Eyes Open and Closed (Firm EO, Firm EC) and standing on Foam with Eyes Open and Closed (Foam EO, Foam EC). Postural sway was measured during the test using the Gait and Balance App, an accelerometery based biomechanical measure of *postural control* collected via an iPhone fixed with a secure belt over the participant’s sacrum. The gait and balance app is a reliable and valid measure of postural control and is sensitive to changes not detected by the m-CTSIB [30].

#### Vestibular function

The function of the semicircular canals (SC) and otolith organs of the inner ear were assessed through video head impulse testing (vHIT) and vestibular evoked myogenic potentials (VEMPs), respectively, for each participant. The vHIT (Natus Medical Inc, Version 4.1) involved high acceleration, unpredictable head turns delivered in the plane of each semicircular canal. Cervical and ocular VEMPs (cVEMPs and oVEMPs, respectively) were recorded using disposable 5 mm Ag/AgCI, Cleartrace snap electrodes connected to a Nicolet Synergy EDx device (Natus Medical Inc, version 22.3.0.21). For cVEMPs, the stimulus was an air-conducted 500 Hz tone-burst (2 ms rise/fall, 123 dB SPL) presented at 5 Hz through insert earphones. Responses to 100 stimuli were recorded from the ipsilateral contracted sternocleidomastoid muscles, band-pass filtered (20–2000 Hz) and averaged. The oVEMPs stimulus was a 1 ms square wave of condensation polarity, amplified to 144 dB FL and delivered at 5 Hz using a Brüel & Kjær 4810 mini-shaker to the upper forehead. Responses to 50 stimuli were recorded infra-orbitally during maximum up-gaze, band-pass filtered (3 to 1000 Hz) and averaged. For both cVEMPs and oVEMPs, the average of two trials was used to determine *response presence*, *peak-peak amplitudes*, *amplitude asymmetry ratios* and *latencies*. For cVEMPs, amplitudes were normalised to account for differences in *baseline muscle activation*; a minimum pre-stimulus contraction of 80 was required before classifying a response as absent.

#### Cognitive measures

Vandenberg Mental Rotation Task (VMRT) was used to measure spatial ability, specifically the ability to mentally manipulate and rotate two- and three-dimensional objects. The task was designed by Vandenberg and Kuse (1978) and is used to evaluate *mental rotation* capabilities, which are associated with visual-spatial processing and the ability to manipulate objects in one’s mind [31]. Participants were presented with pairs of 24 images of three-dimensional objects, where one object is a rotated version of the other. The task required participants to decide whether the objects in the pair are the same or different, despite the rotational orientation of one of the images. This task required participant to rotate the mental representation of the object and match it with the provided options, evaluating their *spatial visualization* and *cognitive flexibility*.

The Design Memory task in the WMS-IV was used to assess participant’s *visual memory*. The task involved memorising geometric designs and later identifying and placing them correctly within a grid. The participants were shown a set of abstract geometric designs that vary in complexity for a brief period, typically lasting around 20 s. After viewing the designs, the participants were asked to select the correct designs from a set of options and place them into a grid. The task required the participant to remember both the specific features of the design and its placement within the grid, testing their *spatial memory* and *visual encoding*.

### MRI data

MRI data collection was conducted at The University of Auckland Centre for Advanced MRI (CAMRI) in Auckland, New Zealand using a 3.0T Siemens MAGNETOM Skyra scanner (Siemens, Germany). Each participant was subjected to a non-contrast MR brain scan using a 20-channel head coil in the headfirst, supine orientation. High-resolution whole-brain T1-weighted images were acquired using the three-dimensional magnetisation-prepared rapid gradient-echo (MP-RAGE) sequencing. The parameters used for the MRI data acquisition are as follows: Slice Thickness = 1.00 millimetres (mm); Field of View = 256 × 256 mm; Number of Acquisitions = 1; Repetition Time (TR) = 2000 milliseconds (ms); Echo Time (TE) = 2.85 ms; Inversion Time = 880 ms; Flip Angle = 8°; Auto Voxel Size = 1.0 × 1.0 × 1.0 mm and Slice Thickness = 1.00 mm.

MRI data that was in the Digital Imaging and Communications in Medicine (DICOM) format, were downloaded and converted to the Neuroimaging Informatics Technology Initiative (NIfTI) format using the “Convert DICOM to NIfTI” tool within the Import menu of the MRIcron image viewer software. NIfTI data were then processed for predicted brain age and brain morphometry analysis.

#### Predicted brain age

The predicted brain age of the participants was calculated using the brainageR software, Version 2.0 (24 Sep 2019) [32], (downloaded from https://github.com/james-cole/brainageR). BrainageR generates a brain-predicted age value from the T1-weighted MRI image, utilizing a Gaussian Processes regression, implemented in R, through the kernlab package. It performs segmentation and normalisation on the T1-weighted MRI image via SPM12 (through MATLAB). The normalised images are loaded into R using the RNfiti package, and the various brain vectors like gray matter (GM), white matter (WM) and cerebrospinal fluid (CSF) are masked and combined. The rotation matrix of the Principal Components (PC) Analysis is applied to the data and 435 PCs are used to predict the brain age value with the trained model in R’s kernlab package.

The brainageR script was run for all the 15 subjects, which created the predicted brain ages for all the subjects. The predicted brain age for all the subjects was collated using the collate_brain_ages.sh utility to combine the individual output.csv files for each subject. Brain age delta was calculated for each participant as the difference between the predicted brain age and the chronological age (predicted brain age - chronological age).

#### Brain morphometry analysis

Brain morphometry analysis was performed on brain regions associated with the hearing, balance, vestibular and cognitive functions. These were identified as Heschl’s Gyrus located in superior temporal gyrus for hearing [33]; cerebellum, basal ganglia, thalamus hippocampus, parietal cortex, and frontal cortex for balance [34, 35]; parieto-insular cortex (i.e. insula and parietal cortex) and superior temporal cortex for vestibular functions [36, 37]; and parietal cortex, temporal cortex, anterior cingulate cortex, pericalcarine cortex and frontal cortex for cognitive measures [38–42]. The brain morphometry analysis was performed as below.

The brain regions of interest (ROIs) were extracted, and various brain attributes (volume, surface area, thickness and curvature) were calculated using FreeSurfer software, Version 7.4.1 (June 2023), (downloaded from https://surfer.nmr.mgh.harvard.edu). FreeSurfer, is a free, open-source software package that uses the T1-weighted MRI data to perform brain segmentation and cortical parcellation, allowing the analysis of brain morphometry quantitatively. The image processing command line pipeline “recon-all” was used to reconstruct a smooth, continuous, two-dimensional cortical surface from three-dimensional volume images. The “recon-all” pipeline extensively processes every image with more than 30 steps involving: motion correction, intensity normalisation, Talairach transformation, skull stripping, brain extraction, atlas registration, gray and white matter volumetric segmentation, white matter and pial surface reconstruction, average curvature mapping, cortical parcellation, and cortical and subcortical region labelling [43]. FreeSurfer ‘parcellates’ the reconstructed cortical gray matter (GM) surface into anatomically distinct ROIs based on structural architecture (myeloarchitecture or cytoarchitecture), functional roles, and connectivity patterns using two different atlases [44]. This study worked with the Desikan-Killiany atlas which parcellates the cortex into a total of 68 ROIs for both hemispheres (34 per hemisphere) [45].

Parcellation files (lhaparc.stats for the left hemisphere, rhaparc.stats for the right hemisphere) and segmentation file (aseg.stats) created by FreeSurfer were used to extract the ROI-based metrics for the identified brain regions, such as: thickness (the distance between the WM i.e. inner boundary of the cortex and pial i.e., the outer boundary of the cortex), surface area (the sum of the areas of all the triangles on the reconstructed cortical surface mesh), mean curvature (how a point on the surface of the cortex is embedded in space) and volume (of the GM between WM and pial) for each subject [43, 46]. The different ROI-based metrics from the parcellation and segmentation files for each subject were then collated to perform statistical analyses for the two groups as outlined in the Statistical analysis.

Structural measurements were also compared between groups using a template that was an average of 40 subjects, the *fsaverage* template, that were combined using the spherical averaging technique [47]. A FreeSurfer Group Descriptor (FSGD) file was created to extract group label covariates, and a contrast matrix was created with Control: -1 and BE: 1. All the individual structural maps were combined into a single dataset and resampled to the *fsaverage* template for volume and thickness for both hemispheres, at 10 mm smoothing using the command *mris_preproc*. Once all the subjects were concatenated into a single dataset, a general linear model was fitted using FreeSurfer’s *mri_glmfit* command. Cluster-wise correction for multiple comparisons was performed, where instead of the voxels, a cluster of voxels were tested for significance, as described by Woo et al., 2014 [48], to highlight significant effects between subjects that may not necessarily align with the standard predefined anatomical regions. The *mri_glmfit_sim* command in FreeSurfer was used to create cluster-corrected maps for thickness and volume measurement for both hemispheres at 10 mm smoothing using Monte Carlo simulation for vertex-wise cluster threshold of -log_10_(1.3) (corresponding to a p-value of 0.05) and a cluster-wise p-threshold of 0.05 for the positive direction.

### Statistical analysis

Statistical analyses were performed on the balance, cognitive assessments, brain age and ROI-based metrics using the Student’s t-test in the RStudio (Version 2024.09.0 + 375 “Cranberry Hibiscus” Release (2024-09-16) for Windows) to test if the difference between the two groups was statistically significant. Vestibular test results were analysed in SPSS (IBM Corp, version 30) using General Linear Mixed Models (GLMM). For each model, the participants ear was included as a repeated measure (Fixed Factor) and the covariance structure was selected based on Akaike’s Information Criterion. Peak head velocity was included as a covariate in the GLMM for vHIT gain. The significance was set at a p-value < 0.05.

For the multiple comparisons, we decided not to employ a False Discover Rate (FDR), in order to minimize type II error (see Hooper, 2025 for a critical discussion) [49–52]. However, effect sizes were calculated (Cohen’s d) in order to provide some idea of the practical significance of the differences. To estimate the standardized mean difference between the two groups, Cohen’s d was computed from the t-statistic, adjusting for unequal group sizes. Effect sizes were interpreted based on established guidelines (small = 0.2, medium = 0.5, large ≥ 0.8). Negative values indicate higher measurements in the BE group, while positive values indicate higher measurements in the control group.

All of the MRI related tests were performed by an assessor who remained blinded until after testing was complete.

## Results

### Participants demographics and activity levels

The study included 15 participants (*n* = 15), aged between 55 and 65. The participants were divided into Control group (*n* = 6) and Balance exercise group (BE) (*n* = 9). There were 6 females and 0 males in the Control group and 5 females and 4 males in the BE group. The chronological age for the Control group was 60.3 ± 2.60 years and for the BE group was 62.3 ± 2.20 years. In the week prior to the assessment, Control group had not partaken in any form of balance exercise while the BE group had spent 3.44 ± 3.09 h doing balance or coordination exercises like yoga, dance, surfing or a balance exercise group class. The control group spent 5.00 ± 2.65 h while BE group spent 8.89 ± 3.31 h in total for active recreation in the week prior to assessment. Of these control group spent 0.20 ± 0.45 h in vigorous activity, 1.70 ± 1.20 h in moderate activity and 3.30 ± 1.64 h in light activity whereas BE group spent 0.77 ± 0.59 h in vigorous activity, 4.89 ± 3.28 h in moderate activity and 2.91 ± 2.03 h in light activity. The difference in the total exercise hours as well as the various levels of activity between the two groups was not statistically significant (*p* > 0.05) except for in the balance exercise hours (*p* = 0.018). Participants in the BE group reported greater active time per week in the previous 12 months and had a more varied range of active recreational activities than the Control group, however this difference was not statistically significant (*p* > 0.05).The demographics and activity levels of the participants are outlined in Table 1.

### Clinical assessments

There was no significant difference in standing postural control between the two groups in any of the four m-CTSIB conditions (*p* > 0.05). No significant group differences were observed in vHIT gain for the horizontal, anterior or posterior semicircular canals (*p* > 0.05). Amplitudes and peak latencies for cVEMPs did not differ significantly between the two groups (*p* > 0.05). There were no significant group or ear differences in oVEMP latencies (*p* > 0.05). However, amplitudes for oVEMPs were significantly higher for the no exercise group in contralateral measurements of the response in the left eye with stimulation of the right ear (*p* = 0.005) and the response in the right eye with stimulation of the left ear (*p* = 0.005). The mental rotation, visual spatial memory tests VMRT and DM (WMS-IV) showed no significant difference between the two groups (*p* > 0.05).

### Brain age

The statistical analysis showed no significant differences for the chronological age, predicted brain age and brain age delta (*p* > 0.05) between the two groups. The results for the predicted brain age and brain age delta for the two groups are shown in Table 1.

**Table 1:** Demographics, activity levels and the results of brainager for the two groups

Demographics	Control Group	Balance Exercise Group
n	6	9
Sex (male/female)	0/6	4/5
Chronological Age (SD)	60.33 (2.60)	62.78 (2.20)
**Group Selection Criterion**		
Balance exercises done for 1 h or more in a week for most weeks in the last 5 years (n)	N/A	Yoga (5), Dance (2), Surfing (1), Balance exercise class (1)
**Hours spent in active recreation in the week prior to assessment**		
Total Exercise Hours, mean (SD)	5.00 (2.65)	8.89 (3.31)
Balance Exercise Hours, mean (SD)	0	3.44 (3.09)
Vigorous Activity Hours, mean (SD)	0.20 (0.45)	0.77 (0.59)
Moderate Activity Hours, mean (SD)	1.70 (1.20)	4.89 (3.28)
Light Activity Hours, mean (SD)	3.30 (1.64)	2.91(2.03)
**Activities done for the purpose of exercise**,** sport**,** or recreation in the previous 12 months**		
Activities done in the last 7 days prior to assessment (n)	Walking (3), Running (1), Gardening (3), Games with children (2)	Walking (8), Gym workout (3), Group exercise class (1), Yoga (5), Dance (2), Golf (1), Surfing (1), Gardening (6), Games with children (1)
Activities done in the last 12 months prior to assessment (n)	Walking (3), Running (1), Group exercise class (1), Dance (1), Cycling (1), Kayaking (1), Gardening (3), games with children (2)	Walking (8), Running (3), Gym workout (4), Group exercise class (2), Yoga (6), Dance (3), Swimming (4), Cycling (3), Overnight tramp (2), Fishing (1), Golf (2), Table tennis (1), Surfing/ body boarding (4), Gardening (8), Games with children (5)
**BraingageR Results**		
Predicted Brain Age (SD)	59.92 (9.03)	64.23 (7.00)
Brain Age Delta (SD)	-0.41 (9.89)	1.45 (6.52)

### Brain morphometry analysis

#### Brain ROIs

**Fig. 1:**
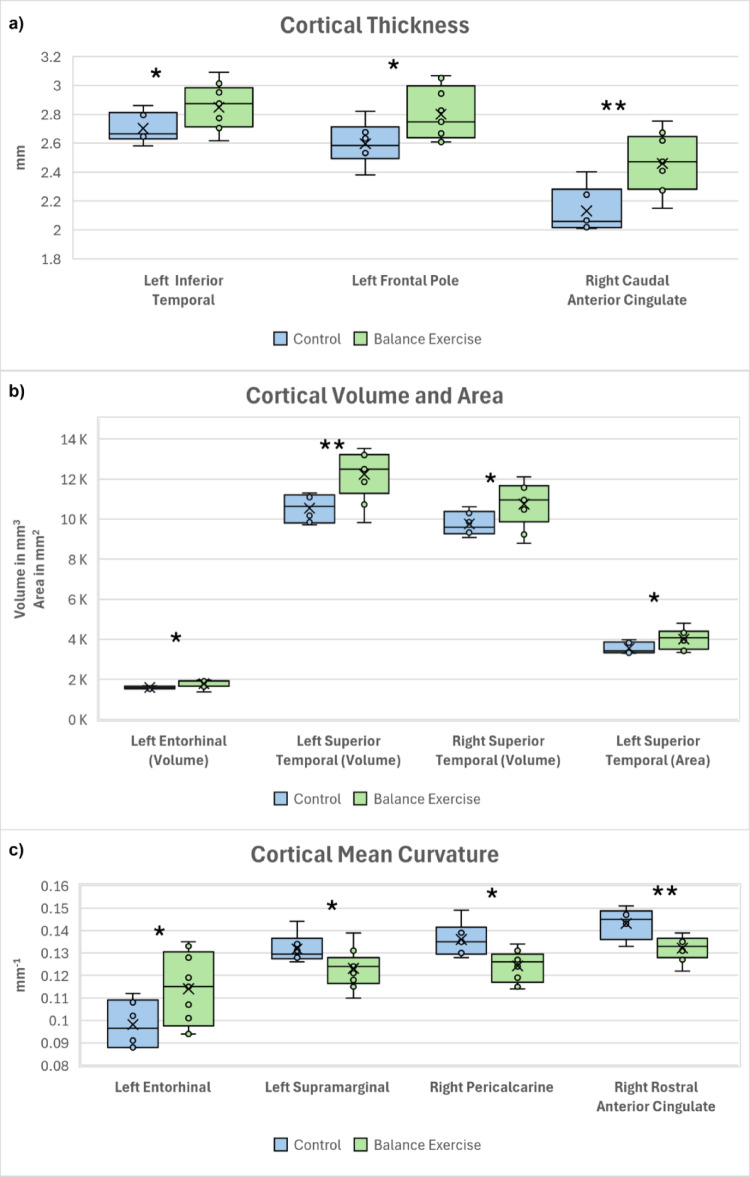
Boxplots with whiskers of statistically significant ROI-based measurements (**a**: thickness, **b**: volume and surface area, **c**: mean curvature) showing the data distribution and variability between the two groups (*, ** for p-values < 0.05, 0.01 respectively)

**Fig. 2:**
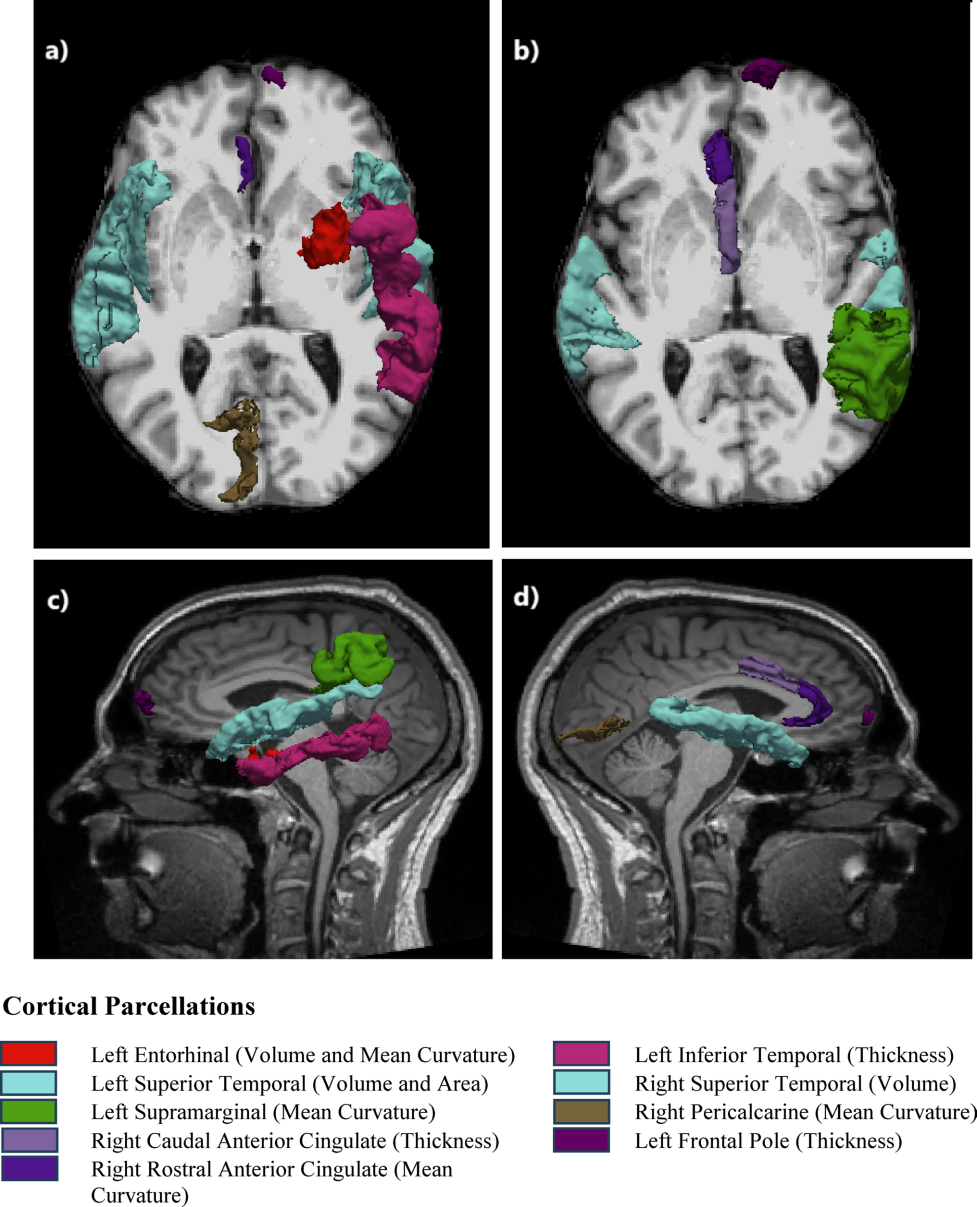
The axial (**a**: inferior, **b**: superior) and sagittal (**c**: left, **d**: right) views of the brain cortical parcellations with statistically significantly different measurements between the two groups shown in colour

The ROI-based statistical analysis of different measurements (thickness, surface area, mean curvature and volume) of cortical parcellations and segmentation, showed 11 measurements to be statistically significant. The mean thickness of the right caudal anterior cingulate, left frontal pole and left inferior temporal cortex for the Control group was 2.13, 2.60 and 2.70 mm, respectively, and for the BE group was 2.46, 2.80 and 2.85 mm, respectively, showing a statistically significant difference of -0.33 mm, -0.20 mm and − 0.15 mm, respectively, with a p-value of 0.004, 0.035 and 0.049, respectively and large effect sizes with Cohen’s d of -1.9, -1.2 and − 1.1, respectively. The mean volume of the left superior temporal, left entorhinal and right superior temporal cortex for the Control group was 10500, 1590 and 9750 mm3, respectively; for the BE group it was 12200, 1780 and 10700 mm3, respectively, showing a statistically significant difference of −1700, -190 and -950mm3, respectively, with a p-value of 0.006, 0.022 and 0.045, respectively and large effect sizes with Cohen’s d of -1.7, -1.4 and − 1.2, respectively. The mean surface area of the left superior temporal cortex for the Control group was 3550 mm2 and for the BE group was 4010 mm2 showing a statistically significant difference of -460 mm2 with a p-value of 0.038 and a large effect size with a Cohen’s d of -1.2. The mean mean-curvature of the right rostral anterior cingulate, right pericalcarine, left entorhinal and left supramarginal cortex for the Control group was  0.143, 0.136, 0.009 and 0.132 mm-1, respectively; for the BE group it was  0.132, 0.124, 0.114 and 0.123 mm-1, respectively, showing a statistically significant difference of  0.011, 0.018, -0.015 and 0.009 mm-1, respectively, with a p-value of 0.009, 0.011, 0.038 and 0.045, respectively and large effect sizes with Cohen’s d of 1.7, 1.6, -1.2 and 1.2, respectively. The results for the statistically significant brain ROI measurements between the two groups are detailed in Table 2. Boxplots with whiskers of statistically significant brain ROI measurements showing the data distribution and variability between the two groups are shown in Fig. 1 (a: thickness, b: volume and surface area, c: mean curvature). Figure 2 shows the axial (a: inferior, b: superior) and sagittal (c: left, d: right) views of the brain ROIs with statistically significantly different measurements between the two groups shown in colour.

**Table 2:** The results for the statistically significant ROI-based parcellation measurements (thickness, surface area, mean curvature and volume) between the two groups (BE: balance exercise group) sorted by p-value (smallest p-value with highest significance shown on the top)

Cortical Measurement	*P* Value	Cohen’s d	T Statistics	Degrees of Freedom	Standard Error	Mean of Control	Mean of BE group	Mean Difference (Control -BE group)	Lower CI (95%)	Upper CI (95%)
Right Caudal Anterior Cingulate Thickness (mm)	0.004	-1.9	-3.541	12.505	0.09	2.13	2.46	-0.33	-0.53	-0.13
Left Superior Temporal Volume (mm^3^)	0.006	-1.7	-3.281	12.79	515.00	10500.00	12200.00	-1700.00	-2800.00	-575.00
Right Rostral Anterior Cingulate Mean Curvature (mm^− 1^)	0.009	1.7	3.29	8.964	0.00	0.14	0.13	0.01	0.0035	0.02
Right Pericalcarine Mean Curvature (mm^− 1^)	0.011	1.6	3.076	10.25	0.00	0.14	0.12	0.02	0.0033	0.02
Left Entorhinal Volume (mm^3^)	0.022	-1.4	-2.664	10.98	71.60	1590.00	1780.00	-190.00	-348.00	-33.10
Left Frontal Pole Thickness (mm)	0.035	-1.2	-2.363	12.255	0.09	2.60	2.80	-0.20	-0.39	-0.02
Left Entorhinal Mean Curvature (mm^− 1^)	0.038	-1.2	-2.308	12.991	0.01	0.10	0.11	-0.02	-0.03	-0.001
Left Superior Temporal Area (mm^2^)	0.038	-1.2	-2.309	12.847	200.00	3550.00	4010.00	-460.00	-892.00	-29.20
Left Supramarginal Mean Curvature (mm^− 1^)	0.045	1.2	2.22	12.627	0.00	0.13	0.12	0.01	0.0002	0.02
Right Superior Temporal Volume (mm^3^)	0.045	-1.2	-2.217	12.706	446.00	9750.00	10700.00	-950.00	-1950.00	-23.10
Left Inferior Temporal Thickness (mm)	0.049	-1.1	-2.175	12.991	0.07	2.70	2.85	-0.15	-0.29	-0.001

#### Cluster based group analysis

**Fig. 3:**
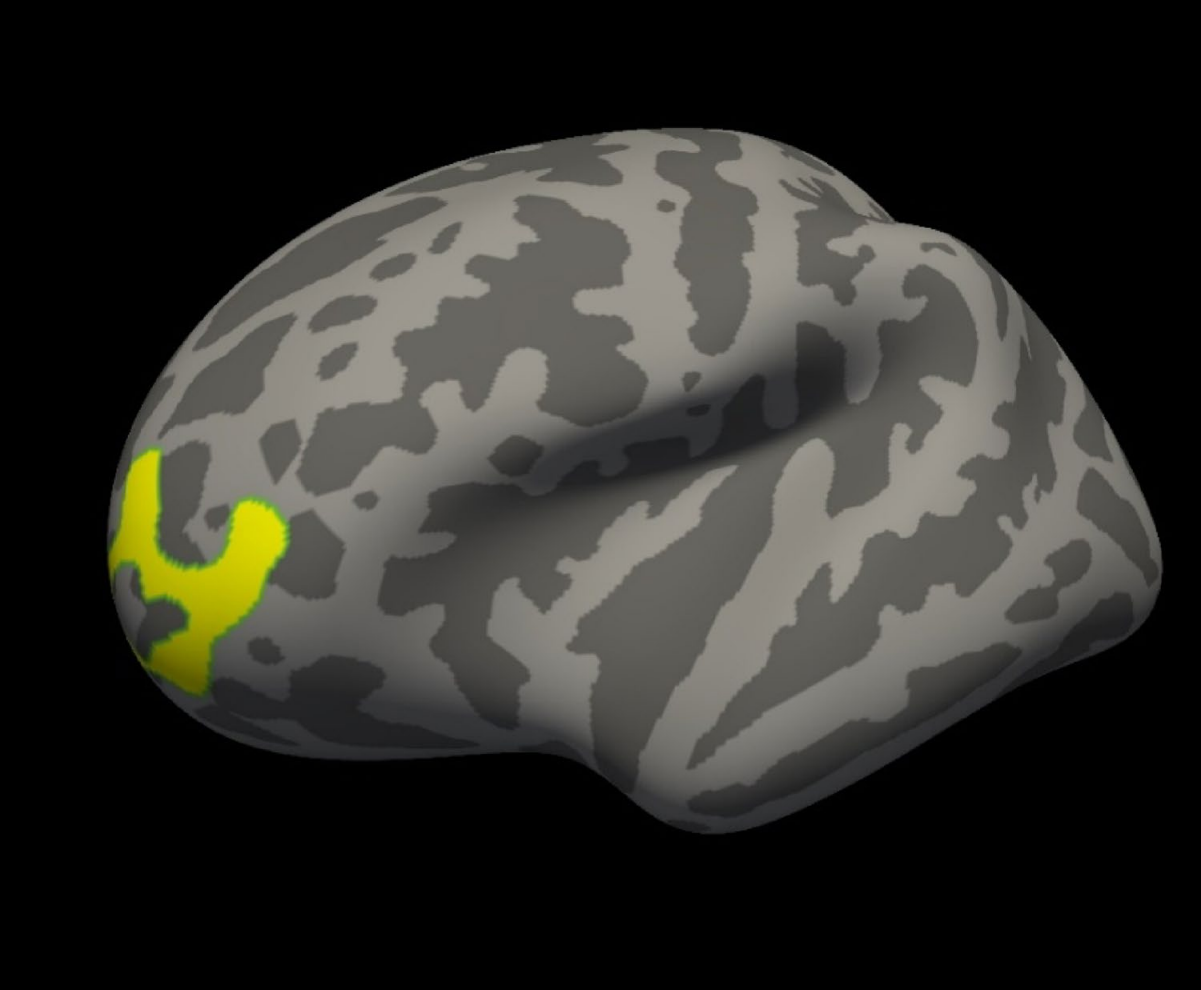
The significant cluster after Cluster wise Correction for Multiple Comparisons, left Rostral Middle Frontal Gyrus, displayed in yellow on the fsaverage template

The results for group wise cluster-corrected maps for thickness and volume measurements for both hemispheres showed one significant cluster for the left hemisphere volume at 10 mm smoothing, a vertex-wise cluster threshold of -log_10_(1.3) (p-value = 0.05) and a cluster-wise p-threshold of 0.05. The significant cluster after cluster-wise correction for multiple comparisons was in left rostral middle frontal gyrus at co-ordinates − 39.0, 47.2, 4.2 and a size of 1196.64 mm^2^. The volume of the left rostral middle frontal gyrus was statistically significantly higher for the BE group with a cluster-wise p-value of 0.036. The significant cluster rendered on the *fsaverage* template is shown in Fig. 3.

## Discussion

### Participants

The study aimed to recruit individuals between the age of 55–65 as this age is at the cusp of cognitive and motor declines [53, 54]. It is the age when people can still choose to have an active lifestyle before their physical strength is diminished and their lifestyle choice of activity can shed a light on the cognitive decline status. The number of participants recruited for the study were restricted due to COVID-19 pandemic lockdowns. This restrained the sample size to a total of 15 with unequal distribution of participant numbers between the groups (*n* = 6 for Control, *n* = 9 for BE group) and inability to recruit any males participants for the Control group. Due to the small sample size the ability to detect true and small to moderate effects was diminished, and increased the likelihood of Type II errors (or false negatives) [55].

### Brain age

We did not observe a result as per the hypothesis (the BE group having younger brains); this could be because of the lower mean chronological age (60.33 for the Controls compared to 62.78 for BE group) and higher variance in the Control group for the predicted brain age (± 9.03 for the Controls compared to ± 7.00 for the BE group) and brain age delta (± 9.89 for the Control group compared to ± 6.52 ).

### Brain morphometry analysis

#### Brain rois

There were 11 cortical parcellation measurements that were statistically significant between the two groups with large effect sizes suggesting biological relevance reflecting group-level modifications in cortical morphology. There were 8 measurements that were higher for the BE group and 3 measurements that were higher for the Control group. All the measurements that were higher in the Control group were for mean curvature. Of the 8 measurements higher in the BE group, 1 was for mean curvature, 1 for surface area, 3 for volume and 3 for thickness. As the brain undergoes cortical atrophy and shape change with age, various brain attributes change such as the: brain volume shrinks [56], surface area and thickness decrease in certain brain regions [57] and there is a general decrease in the sulcal mean curvature [58]. Accordingly, the effect of age is seen more prominently for certain brain attributes in the Control group than in the BE group.

The cortical volumes of the left and right superior temporal and left entorhinal were statistically significantly higher in the BE group compared to the Control group. The superior temporal region, an integral part of the temporal lobe, is critical for processing of the language, speech and auditory inputs and in social perception and cognition by interpretating facial expressions, emotions and intentions of others [59, 60]. The left entorhinal cortex, located in the medial temporal lobe, is important for cognitive processes like learning and memory, spatial navigation and sensory perception [61, 62]. With normal aging, the volume of the superior temporal and entorhinal cortex tends to shrink gradually [63]. A decrease in superior temporal volume is often seen in patients with schizophrenia and is usually associated with memory impairment and dysfunction of language and auditory processing [64]. The volume of the left entorhinal cortex is often studied because it is one of the earliest regions to show structural changes in neurodegenerative diseases such as Alzheimer’s disease [65, 66]. A decrease in entorhinal volume, is often associated with memory impairments, cognitive decline and difficulties in learning new information [67, 68]. The higher volumes of the left entorhinal cortex and superior temporal as seen in the BE group in this study indicates the beneficial effect of balance exercise on safeguarding the learning and cognitive abilities.

The cortical thickness of the left superior temporal, right caudal anterior cingulate and left frontal pole were statistically significantly higher in the BE group compared to the Control group. The caudal anterior cingulate is a subregion of the anterior cingulate, located in the medial frontal lobe of the brain and plays a crucial role in social cognition like conflict monitoring, error detection, and attention [69] and visuospatial and memory functions [70]. The left frontal pole is located at the most anterior part of the left frontal lobe and involved in high-level cognitive functions like problem-solving, abstract thinking, future planning and decision-making [71, 72]. With age, there is thinning of the superior temporal, caudal anterior cingulate and left frontal pole cortex [73–75]. Reduced cortical thickness of the left frontal pole is seen in patients with bipolar depression and Parkinson’s disease and can influence behaviour and cognitive functions [76, 77]. A thinning of the left superior temporal region is seen in neurodegenerative disorders like dementia and aphasia and is associated with cognitive decline [78]. A decrease in thickness of the right caudal anterior cingulate is seen in patients with multiple sclerosis and is associated with figural fluency [79]. The greater thickness of the left superior temporal, right caudal anterior cingulate and left frontal pole regions seen in the BE group in this study demonstrates the positive contribution of balance exercise on protecting the memory and cognitive functions.

The surface area of the left superior temporal lobe was statistically significantly higher in the BE group compared to the Control group. The temporal lobe shows a decrease in surface area with age [80]. A smaller superior temporal gyrus is seen in patients with schizophrenia and can affect social cognition, auditory perception, language processing, and emotional regulation [81]. The higher surface area of the left superior temporal region in the BE group seen in this study indicates an advantageous impact of balance exercise on mental health.

The mean curvature of the left entorhinal cortex was statistically significantly higher in the BE group compared to the Control group. However, the mean curvature for the right rostral anterior cingulate, right pericalcarine and left supramarginal was statistically significantly higher in the Control group compared to the BE group. With age, as the brain undergoes cortical atrophy, there is reduced mean curvature of sulci as these structures flatten and widen [82]. However, the mean curvature of brain gyri increases with age making them more curved [82]. An increased cortical curvature is also seen in patients with multiple sclerosis due to atrophy to the underlying white matter [83]. Changes in cortical mean curvature and folding patterns is seen to disrupt neural connectivity and efficient neural processing in schizophrenia [84]. The increased mean curvature of brain regions seen in the Control group but not in the BE group in this study suggests a favourable influence of balance exercise on preserving the neural connectivity.

#### Cluster based group analysis

After cluster-wise correction for multiple comparisons, the volume of the left rostral middle frontal gyrus cluster was statistically significantly higher for BE group than the Control group. The rostral middle frontal gyrus is a region in the frontal lobe and is part of the prefrontal cortex, that is associated with higher brain functions including decision-making, problem-solving, planning, organizing, motivation, discipline, and emotional regulation [85]. The rostral middle frontal gyrus is critical for higher-order executive functions related to stress perception and appraisal, including attention, working memory, planning, executive cognition, and emotion regulation [86]. The dominant (left) middle frontal gyrus plays a key role in the development of literacy, while the nondominant (right) middle frontal gyrus is responsible for numeracy [87]. The higher volume of the left rostral middle frontal gyrus seen in the BE group in this study proposes the progressive change by balance exercise for preserving the executive cognitive abilities.

Previous studies have demonstrated that age-related white matter degeneration and cortical atrophy may be more pronounced in males, particularly within left frontal and temporal cortices [88, 89]. The presence of increased prefrontal volume in a BE group that included both sexes is noteworthy. Although causal inferences cannot be drawn with this observational study, these findings suggest that balance exercise may contribute to the preservation of executive-control regions that are critical for maintaining cognitive resilience in later life.

### Clinical functional assessments

While there were adverse changes in the bran ROIs associated with balance, vestibular and cognitive functions in the Control group that were not seen in the BE group, changes in these functions were not observed in the associated clinical assessments. Though this snapshot in time cannot prove a protective influence of balance exercise on the brain, it opens potential for an interesting avenue of research in studies with a longer follow up period. It is possible that changes in brain volume and other measurements precede clinical changes,, but further longitudinal studies are required to understand whether structural brain changes may translate to future clinical benefits. It is also possible that the balance exercises help to compensate functionally without altering the raw clinical scores especially the vestibular reflex strength that was seen to be higher in the control group. Cognitive declines seen in aging individuals could be evaded or delayed with balance exercise because of its advantageous influence on brain areas like the left entorhinal cortex, left frontal pole, left superior temporal, and rostral middle frontal gyrus that are associated with cognitive functions, and show a statistically significant higher values in the BE group compared to the Control group. Similarly, declines in memory can be slowed down with balance exercise due to its progressive effect on brain regions like the left entorhinal cortex, left and right superior temporal area, caudal anterior cingulate, and rostral middle frontal gyrus that are associated with memory, and show statistically significant higher values in the BE group compared to the Control group.

The neurostructural regions seen to be statistically significantly larger in the BE group could be responsible for promoting healthy aging, protecting the deterioration of motor, memory and cognitive skills in the elderly. The results of this study demonstrate that balance exercise may have the potential to attenuate age-linked functional decays and enhance brain health in the ageing population.

### Limitations of the study

There were several challenging factors that compromised and limited the generalisability of this study. The lockdowns imposed during the COVID-19 pandemic made it difficult to recruit participants for the study, restricting the study to a small sample size (*n* = 15) with unequal sample sizes between the groups (*n* = 6 for Control, *n* = 9 for BE group), and no male participants recruited for the Control group. The small, unequal and sex-imbalanced sample significantly limited the generalizability and sensitivity of the study. The sex-imbalanced sample increased the likelihood of Type I errors, where a false difference could have been found because of the sex-imbalance rather than the condition. Given the well-documented sex differences in brain structure and age-related brain atrophy, the sex imbalanced samples in this study represents a limitation of the study. Future studies should use sex-matched cohorts or include sex as a covariate to clarify the independent effects of balance exercise. The small sample size increased the likelihood of Type II errors limiting the study to finding only large differences or large “effects” between the two groups. Additionally, the variance in the smaller group (Control group in this study) probably skewed the means disproportionately, minimizing effects and reducing generalizability of the study results to a wider population. Given the exploratory nature of this pilot study, findings should be interpreted as hypothesis-generating rather than confirmatory. For future studies, the statistical power calculations should be considered to guide participants recruitment numbers.

The groups for the study were divided based on the self-reported activity level through questionnaire rather than monitored objective measurements, which can potentially introduce recall bias and may bring in a degree of unreliability and grouping prejudice. Moreover, the study focused on data from a single observational time-point looking at differences between the groups based on existing active lifestyles instead of evaluating the effect of exercise intervention.

### Future research directions

The data from this study have given us an insight into the effects of long-term balance exercise on brain health. The changes seen suggest that balance exercise could help maintain cerebral volume in critical areas. Future research could involve longitudinal studies that determine whether this increase in brain volume delays the clinical signs of aging in terms of balance, vestibular and cognitive function. Future studies should validate the effects of the balance exercise on a larger and more diverse cohort that would represent the population more accurately. We could also extend the scope of the study beyond the structural MRI and include fMRI to study the functional connectivity of different regions of the brain and Diffusion Tensor MRIs to study and map the nerve tracts and structure of the white matter of the brain. A correlation analysis between the MRI measurements and vestibular function and cognitive measures can also be conducted to uncover any possible associations between changes in neural structures and balance, spatial orientation, and cognitive health. This would considerably enhance the understanding of how exercise contributes to preserving cognitive, motor and memory functions during healthy aging.

## Conclusion

This study examined the relationship between balance exercise and healthy aging by quantifying various brain attributes using T1-weighted structural brain MRI data. There were no significant differences found between the BE group and Control group in the clinical measurements and brain age. The BE group showed statistically significantly higher values with large effect sizes for the left and right superior temporal volume, left superior temporal area, left inferior temporal thickness, left entorhinal volume and mean curvature, right caudal anterior cingulate thickness and left frontal pole thickness. These findings, while statistically significant and associated with large effect sizes, should be interpreted with caution given the sample size and exploratory nature of the study. The left rostral middle frontal gyrus was the statistically significant cluster after correction for multiple comparisons. The brain regions seen to be larger in the BE group are associated with functions like memory formation, responses to stress, decision making, focus, attention, mental health and cognitive control. The neurological changes seen in this study could be responsible for healthy aging, protecting or delaying the decline in motor, memory and cognitive capabilities. This study suggests that balance exercise may offset age-related loss of brain volume, surface area and thickness, and improve brain health in the ageing population. It is possible that these brain changes precede clinically observable changes in balance, cognitive and vestibular function, but further longitudinal studies are required to understand whether structural brain changes brought about by low-impact balance and coordination exercise training may translate to future clinical benefits.

## Supplementary Information

Below is the link to the electronic supplementary material.

Supplementary Material 1

Supplementary Material 2

## Data Availability

Data is provided in the supplementary information files.
